# Genetic Characterization of a Panel of Diverse HIV-1 Isolates at Seven International Sites

**DOI:** 10.1371/journal.pone.0157340

**Published:** 2016-06-17

**Authors:** Bhavna Hora, Sheila M. Keating, Yue Chen, Ana M. Sanchez, Ester Sabino, Gillian Hunt, Johanna Ledwaba, John Hackett, Priscilla Swanson, Indira Hewlett, Viswanath Ragupathy, Sai Vikram Vemula, Peibin Zeng, Kok-Keng Tee, Wei Zhen Chow, Hezhao Ji, Paul Sandstrom, Thomas N. Denny, Michael P. Busch, Feng Gao, on behalf of the REDS-III and EQAPOL programs

**Affiliations:** 1 Duke Human Vaccine Institute and Department of Medicine, Duke University Medical Center, Durham, North Carolina, United States of America; 2 Blood Systems Research Institute, San Francisco, California, United States of America; 3 Department of Laboratory Medicine, University of California, San Francisco, California, United States of America; 4 Instituto de Medicina Tropical, Sao Paolo Brazil; 5 National Institute of Communicable Diseases, Johannesburg, South Africa; 6 Abbott Laboratories, Infectious Diseases Research, Abbott Park, Illinois, United States of America; 7 Center for Biologics Evaluation and Research, Food and Drug Administration, Silver Springs, Maryland, United States of America; 8 Institute of Blood Transfusion, Chinese Academy of Medical Sciences, Chengdu, China; 9 Centre of Excellence for Research in AIDS, Department of Medical Microbiology, Faculty of Medicine, University of Malaya, Kuala Lumpur, Malaysia; 10 National HIV & Retrovirology Laboratories at JC Wilt Infectious Diseases Research Center, Public Health Agency of Canada, Winnipeg, Canada; University of Athens, Medical School, GREECE

## Abstract

HIV-1 subtypes and drug resistance are routinely tested by many international surveillance groups. However, results from different sites often vary. A systematic comparison of results from multiple sites is needed to determine whether a standardized protocol is required for consistent and accurate data analysis. A panel of well-characterized HIV-1 isolates (N = 50) from the External Quality Assurance Program Oversight Laboratory (EQAPOL) was assembled for evaluation at seven international sites. This virus panel included seven subtypes, six circulating recombinant forms (CRFs), nine unique recombinant forms (URFs) and three group O viruses. Seven viruses contained 10 major drug resistance mutations (DRMs). HIV-1 isolates were prepared at a concentration of 10^7^ copies/ml and compiled into blinded panels. Subtypes and DRMs were determined with partial or full *pol* gene sequences by conventional Sanger sequencing and/or Next Generation Sequencing (NGS). Subtype and DRM results were reported and decoded for comparison with full-length genome sequences generated by EQAPOL. The partial *pol* gene was amplified by RT-PCR and sequenced for 89.4%-100% of group M viruses at six sites. Subtyping results of majority of the viruses (83%-97.9%) were correctly determined for the partial *pol* sequences. All 10 major DRMs in seven isolates were detected at these six sites. The complete *pol* gene sequence was also obtained by NGS at one site. However, this method missed six group M viruses and sequences contained host chromosome fragments. Three group O viruses were only characterized with additional group O-specific RT-PCR primers employed by one site. These results indicate that PCR protocols and subtyping tools should be standardized to efficiently amplify diverse viruses and more consistently assign virus genotypes, which is critical for accurate global subtype and drug resistance surveillance. Targeted NGS analysis of partial *pol* sequences can serve as an alternative approach, especially for detection of low-abundance DRMs.

## Introduction

One hallmark of HIV-1 characteristics is its extraordinary genetic variability. HIV-1 is classified into four groups (M, N, O and P) [1–4]. HIV-1 group M viruses, which are responsible for most infections in the world can be further classified into subtypes (A, B, C, D, F, G, H, J and K), sub-subtypes (A1-A4 and F1-F2), circulating recombinant forms (CRFs) and countless unique recombinant forms (URFs) [5, 6]. These recombinant viruses account for at least 20% of HIV infections in recent studies [7]. Therefore, the high level genetic diversity of human immunodeficiency virus type I (HIV-1) poses significant public-health and clinical challenges, including implications for performance of blood donor screening, diagnostic testing and patient monitoring by viral load and drug resistance testing [1, 8–10]. Determination of viral loads in HIV-1 infected individuals is critical for prognosis and monitoring the efficacy of antiviral therapy. The cross-subtype reactivity of nucleic acid tests (NATs) for measuring viral loads of different subtypes has been significantly improved in recent years. Although some assays show better detection efficiency for different HIV-1 subtypes and even among different HIV-1 groups [11, 12] the sensitivity of some NATs is still affected by different subtypes [12, 13]

Distinct prevalence of drug resistance mutations (DRMs) and kinetics of DRM emergence were observed among HIV-1 subtypes [14–18], although other studies indicated that outcomes of antiretroviral therapies (ART) may not be affected by subtypes and differences in virological and immunologic responses to ART might be contributed by ethnicity and/or adherences [19–21]. Drug resistance mutations (DRMs) are a major cause of antiretroviral therapy failure, and hence surveillance for the emergence of drug resistance among all subtypes and recombinants has been implemented in ART programs. The worldwide effort to improve treatment outcomes and reduce transmission of HIV-1 through early and optimal delivery of ART and HIV prevention programs should be a coordinated effort that includes national, regional, and global evaluations of HIV-1 drug resistance [22, 23].

The Recipient Epidemiology and Donor Evaluation Study-III (REDS-III) is a multicenter transfusion safety research program, launched in 2011 by the National Institute of Heart, Lung, and Blood of the National Institutes of Health, USA [24]. It is intended that REDS-III serve as an impetus for more widespread recipient and linked donor-recipient research in the United States and other international locations. It includes a domestic component and three distinct international programs in Brazil, China, and South Africa. The REDS-III portfolio includes molecular surveillance of HIV-1 infections in donors [25–27] which necessitated comparison, training and adoption of improved sequencing methods for the partial *pol* gene and/or HIV-1 whole genome to enable high yield of sequence data, better drug resistance classification, and subtype assignment.

Many international surveillance groups, including the REDS-III network, are involved in HIV-1 genetic analyses but in the absence of standardized protocols and external quality assurance these studies may report inconsistent results [28, 29]. The REDS-III and the External Quality Assurance Program Oversight Laboratory (EQAPOL) programs determined that a systematic comparison of results from multiple sites was needed to determine if standardized protocols are required for consistent reports and accurate data analysis. Therefore, the REDS-III and EQAPOL networks planned a multi-center comparison study to assess the molecular methods for accurately determining genotype and testing for DRMs in international reference laboratories. The aim of this study was to compare results of the partial or full *pol* gene sequences generated with various RT-PCR conditions and sequencing methods at seven different international laboratories.

## Materials and Methods

### Assembly of a panel of diverse HIV-1 isolates

A large panel of HIV-1 isolates representing HIV-1 groups, subtypes, CRFs and URFs from different countries was assembled at EQAPOL. The HIV-1 strains were derived from either plasma samples or established isolates and expanded to high titer stocks by short-term culturing with peripheral blood mononuclear cells (PBMC) from HIV-1 negative donors [30]. All virus donations to EQAPOL were collected with ethical review and approval of the collection protocol and informed consent as previously established for the donor organization’s collection activities. This study was approved by the Institutional Review Board (IRB) at Duke University under the IRB exemption protocol from 45 CFR 46. Before previously acquired samples were delivered to Duke University, all samples were de-identified under IRB exemption and no identifiable subject information was provided. The same de-identified virus isolates were sent to evaluation sites for further analysis. Near full-length genome sequences were obtained for all viruses by single genome amplification (SGA) at EQAPOL [30]. The subtype or recombination pattern of each virus was determined by phylogenetic tree analysis (Neighbor-joining method with Kimura two-parameter model), the REGA subtyping tool version 2 (http://jose.med.kuleuven.ac.be/genotypetool/html/subtypinghiv.html) and the jumping profile Hidden Markov Model (jpHMM) tool (http://jphmm.gobics.de/) [31]. If discordant subtyping results were observed for any regions in a sequence among different algorithms, phylogenetic trees were constructed for this particular region to determine the subtype origin of this region of sequence.

Fifty well-characterized diverse HIV-1 isolates from 20 countries were selected to establish the EQAPOL Genetic Diversity Panel. This 50-member panel consists of 28 viruses from six subtypes and two sub-subtypes (A, B, C, D, F1, F2 and G), 10 from six CRFs (CRF01_AE, CRF02_AG, CRF04_cpx, CRF14_BG, CRF24_BG, and CRF47_BF), 9 URFs and three group O viruses. Each virus was diluted into HIV-1 negative defibrinated plasma (Gemini Bio-Product, Sacramento, CA) to a concentration of 10^7^ viral RNA copies/ml and 1 mL cryopreserved aliquots were coded before they were sent out to test sites.

### International evaluation sites

Seven sites participated in this study. Three of these sites are a part of the international component of REDS-III initiative: the South African National Blood Services (SANBS) in collaboration with the National Institute of Communicable Diseases, South Africa; the Instituto de Medicina Tropical, Brazil; and the Institute of Blood Transfusion, Chinese Academy of Medical Sciences, China. The other four sites were Abbott Laboratories, USA (USA_Abbott); the Laboratory of Molecular Virology at the US Food and Drug Administration, USA (USA_FDA); the University of Malaya, Malaysia; and the National HIV & Retrovirology Laboratories, Public Health Agency of Canada, Canada.

### Subtype determination and detection of drug resistance mutations

Each test site received the same blinded panel of 50 viruses for evaluation and used their own protocol for RT-PCR amplification, sequencing the PCR amplicons, determination of subtypes and detection of major and minor DRMs (S1 Table). Genotypes and DRMs were determined with partial *pol* sequences (protease and partial reverse transcriptase) at six sites (Brazil, South Africa, USA_Abbott, China, Malaysia and Canada) or with complete *pol* sequences generated by NGS at the USA_FDA site. All 50 viruses were analyzed by both Sanger sequencing and NGS (MiSeq, Illumina, San Diego, CA) methods at the Canada site. Raw reads were analyzed using HyDRA, a proprietary data analytical pipeline developed for NGS-based HIV drug resistance (DR) analysis (http://hydra.canada.ca) [32]. To generate *pol* gene consensus sequences at the USA_FDA site, raw reads for the *pol* gene were imported to High-performance Integrated Virtual Environment (HIVE; https://hive.biochemistry.gwu.edu/) and an algorithmic pipeline was used to generate consensus sequences obtained by NGS. Using genotype reference sets from the Los Alamos HIV-1 sequence database (http://www.hiv.lanl.gov) and the hexagon tool in HIVE, reference mapping was performed. Raw reads were assembled with the clonal assembly tool in HIVE. Consensus sequences were generated by linking quasispecies clones for further downstream analysis.

### Comparison of results from all sites

Results (sequences, genotypes and DRMs) for the blinded panels from each test site were reported back to EQAPOL. The results were decoded and compared to near full-length reference sequences generated at EQAPOL. Sequences from each site were aligned to the EQAPOL reference sequences using CLUSTAL W and manually adjusted for optimal alignments with Seaview [33]. If the site subtype assignment and sequence matched the EQAPOL reference sequence, no further analysis was performed. When the site sequences differed from the EQAPOL references, further analysis was carried out to identify the causes for the differences. Subtyping results from each site were categorized as concordant or discordant relative to EQAPOL results. The major and minor DRMs from each site were also compared with those identified by analysis of the EQAPOL sequences. If the reported DRMs were discordant, sequences were further examined for mutations or ambiguous bases at the affected codons.

### GenBank accession numbers

GenBank accession numbers of near full length HIV sequences are: JX140645—JX140653, JX140655—JX140663, JX140665, JX140668, JX140670—JX140672, JX140675, JX140677—JX140679, KC473825, KC473827, KC473836 -KC473840, KC473842—KC473846, KC596061, KC596066—KC596069, KC596071—KC596073, KF859742—KF859744.

## Results

### Majority of group M viruses were amplified at all sites

**Fig 1:**
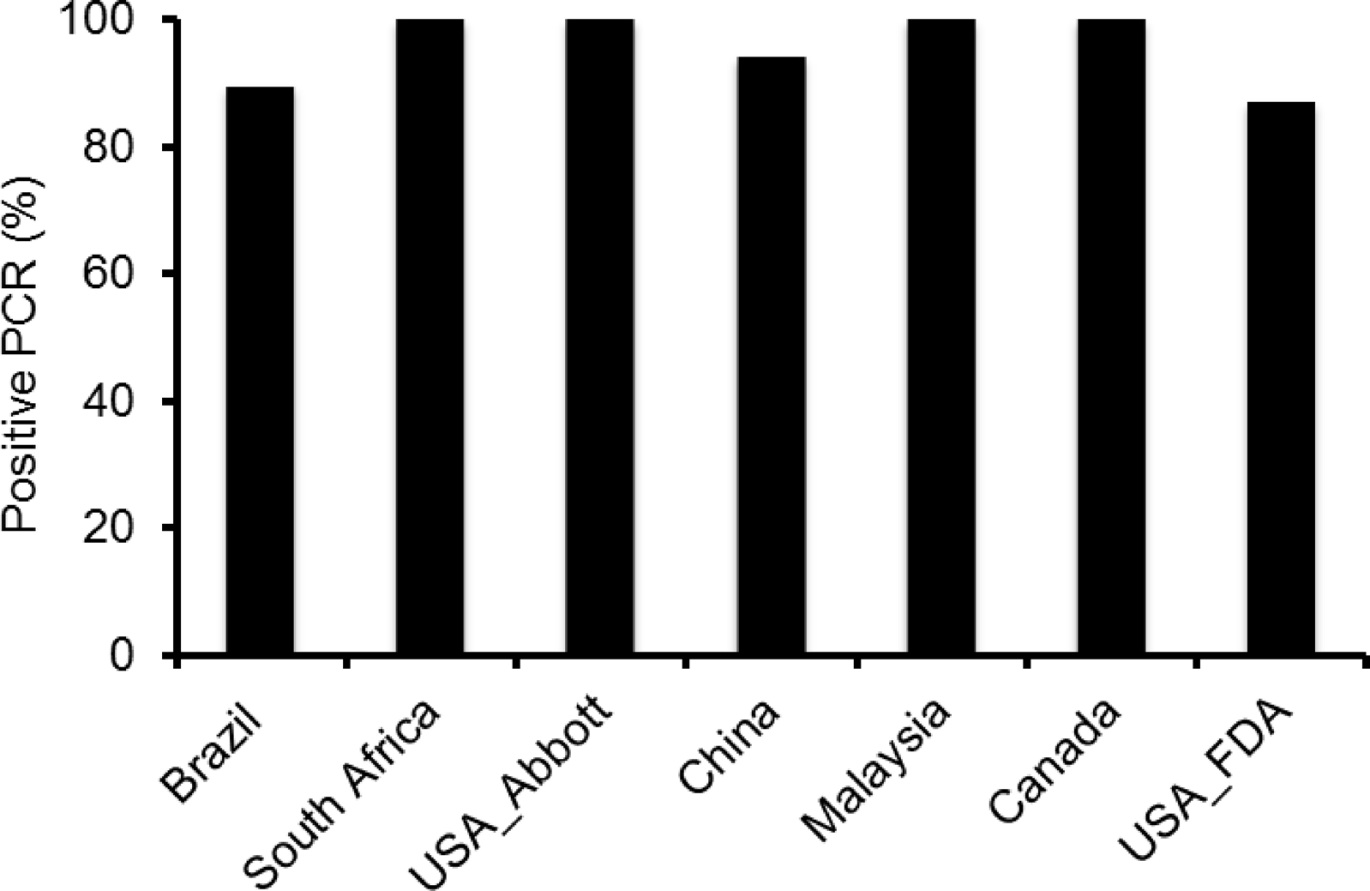
Comparison of PCR success rates. Partial *pol* genes were amplified for 47 group M viruses at six sites. The whole *pol* gene was amplified at the USA_FDA site.

The partial *pol* gene was successfully amplified for all 47 group M viruses at the South Africa, USA_Abbott, Malaysia and Canada sites. It was amplified from 42 and 44 group M viruses at the Brazil and China sites, respectively. All failed PCR amplifications were from independent HIV-1 isolates among all sites except DE00400GR002 that was not amplified at both Brazil and China sites (Table 1), suggesting the PCR negative results were not strain specific. Complete *pol* gene sequences were obtained from 41 samples generated by NGS at the USA_FDA site. All three group O viruses were amplified only at the Malaysia site when an additional PCR primer set specific for group O viruses was used, according to their standard protocol for samples that fail to amplify with group M primers (Table 1). The overall successful RT-PCR amplification rate among six sites that amplified the partial *pol* gene averaged 95.7% (range from 89.4% to 100%) for group M viruses (Fig 1).

**Table 1:** Comparison of subtyping results from all sites.

Sample Name	EQAPOL		Brazil	South Africa	USA_Abbott	China	Malaysia	Canada	USA_FDA ^#^
	Full length genome	Partial *pol*							
DEMA105TZ001	A1	A1	**CRF01**	A1	A1	A1	**CRF02**	A1	Neg
DEMA106ES002	A1	A1	A1	A1	A1	A1	A1	A1	**A1 (AF1)**
DEMB03JP004	B	B	B	B	B	B	B	B	B
DEMB05FR001	B	B	B	B	B	B	B	B	B
DEMB08ES001	B	B	B	B	B	B	B	B	B
DEMB08UY001	B	B	B	B	B	B	B	B	B
DEMB09BO001	B	B	Neg	B	B	B	B	B	B
DEMB09CN002	B	B	B	B	B	B	B	B	B
DEMB09US002	B	B	B	B	B	B	B	B	B
DEMB10CN002	B	B	B	B	B	B	B	B	B
DEMB10ES002	B	B	B	B	B	B	B	B	B
DEMB10ES003	B	B	B	B	B	B	B	B	Neg
DEMB10US001	B	B	B	B	B	B	B	B	Neg
DEMB10US004	B	B	B	B	B	B	B	**B/CRF01**	**B (BD)**
DEMB10VE001	B	B	B	**B,D**	B	B	B	B	B
DEMBXXDE001	B	B	B	B	B	B	B	B	B
DEMBXXPL001	B	B	Neg	B	B	B	B	B	B
DEMC06ES003	C	C	C	C	C	C	C	C	**C (CB)**
DEMC07AO001	C	C	C	C	C	C	C	C	**C (CF1)**
DEMC07BR003	C	C	C	C	C	C	C	C	**C (CBF1)**
DEMC08NG001	C	C	C	C	C	C	C	C	Neg
DEMC09ZA009	C	C	C	C	C	Neg	C	C	C
DEMD07UG002	D	D	D	D	D	D	**CRF10**	D	D
DEMD08UG001	D	D	D	D	D	D	D	D	D
DEMD10CM009	D	D	D	D	D	D	D	D	D
DEMF110ES001	F1	F1	F1	F1	F1	F1	F1	F1	**F1 (F1BD)**
DEMF210CM001	F2	F2	**B/F**	F2	F2	F2	F2	F2	**F2 (F2A)**
DEMG09ES002	G	G	G	G	**CRF14**	G	G	G	Neg
DE00109CN003	CRF01	A1	CRF01	CRF01	CRF01	CRF01	CRF01	CRF01	CRF01
DE00208CM004	CRF02	A1G	CRF02	A1,G	CRF02	CRF02	**DG**	CRF02	CRF02
DE00208CM001	CRF02	A1G	CRF02	A1,G	CRF02	CRF02	CRF02	CRF02	CRF02
DE00206AO001	CRF02	A1G	CRF02	A1,G	CRF02	CRF02	CRF02	CRF02	**CRF02 (02B)**
DE00400GR002	CRF04	A1FGK	Neg	CRF04	CRF04	Neg	CRF04	CRF04	**A1FK**
DE01405BR001	CRF14	G	G	G	*CRF14*	*CRF14*	*CRF14*	G	**BF (BFGDA1F1)**
DE01405ES002	CRF14	G	G	G	*CRF14*	*CRF14*	*CRF14*	*CRF14*	**CRF14_BG (BGD)**
DE02408ES002	CRF24	BG	Neg	*CRF24*	*CRF24*	*CRF24*	*CRF24*	*CRF24*	BG
DE04708ES003	CRF47	BF1	**B**	B,F	*CRF47*	*CRF47*	*CRF47*	*CRF47*	BF1
DE04708ES004	CRF47	BF1	**B**	B,F	*CRF47*	*CRF47*	*CRF47*	*CRF47*	BF1
DEURF09ES005	URF_A1B	B	B	B	B	A1B	B	B	B
DEURF10US008	URF_A1B	B	B	B	B	A1B	B	B	B
DEURF07UG006	URF_A1D	D	D	D	D	A1D	D	D	D
DEURF09GQ001	URF_A1DG	G	G	G,A*	**URF_G/CRF02**	A1DG	G	G	Neg
DEURF07BR002	URF_BC	C	C	C	C	Neg	**CD**	C	**BC**
DEMBF09ES003	URF_BF1	F1	F1	F1	F1	BF1	**CRF72**	F1	BF1
DEMBF09ES006	URF_BF1	BF1	**D/B**	B,F1	BF1	BF1	*CRF12*	*CRF12*	BF1
DEURF07ES002	URF_02/A3	A3	Neg	**A1**	A3	**URF_02/A1**	**A1**	**B**	**URF_02/A1**
DEURF10DZ001	URF_02/06/01	CRF06	**CRF02**	CRF06	CRF06	**U**	**CRF02**	**CRF02**	**URF_AGKD**
DEOXXDE004	O	O	Neg	Neg	Neg	Neg	O	Neg	Neg
DEOXXES001	O	O	Neg	Neg	Neg	Neg	O	Neg	Neg
DEOXXUS001	O	O	Neg	Neg	Neg	Neg	O	Neg	Neg

Subtype results provided by sites were compared to those based on the near full length HIV-1 genome sequences generated by EQAPOL. Negative PCR is indicated as “Neg”, discordant subtypes from sites are indicated in bold, and subtype results that are correctly obtained for CRFs based on the partial *pol* sequences are indicated in italics. Subtype results that are discordant for the partial *pol* gene but concordant for near full-length genome are underlined. Additional subtype fragments are shown in parentheses.

#: Complete *pol* gene sequence.

*: longer 3’ end sequence that contains subtype A region.

### Majority of subtyping results were in agreement with the EQAPOL reference sequences

**Fig 2:**
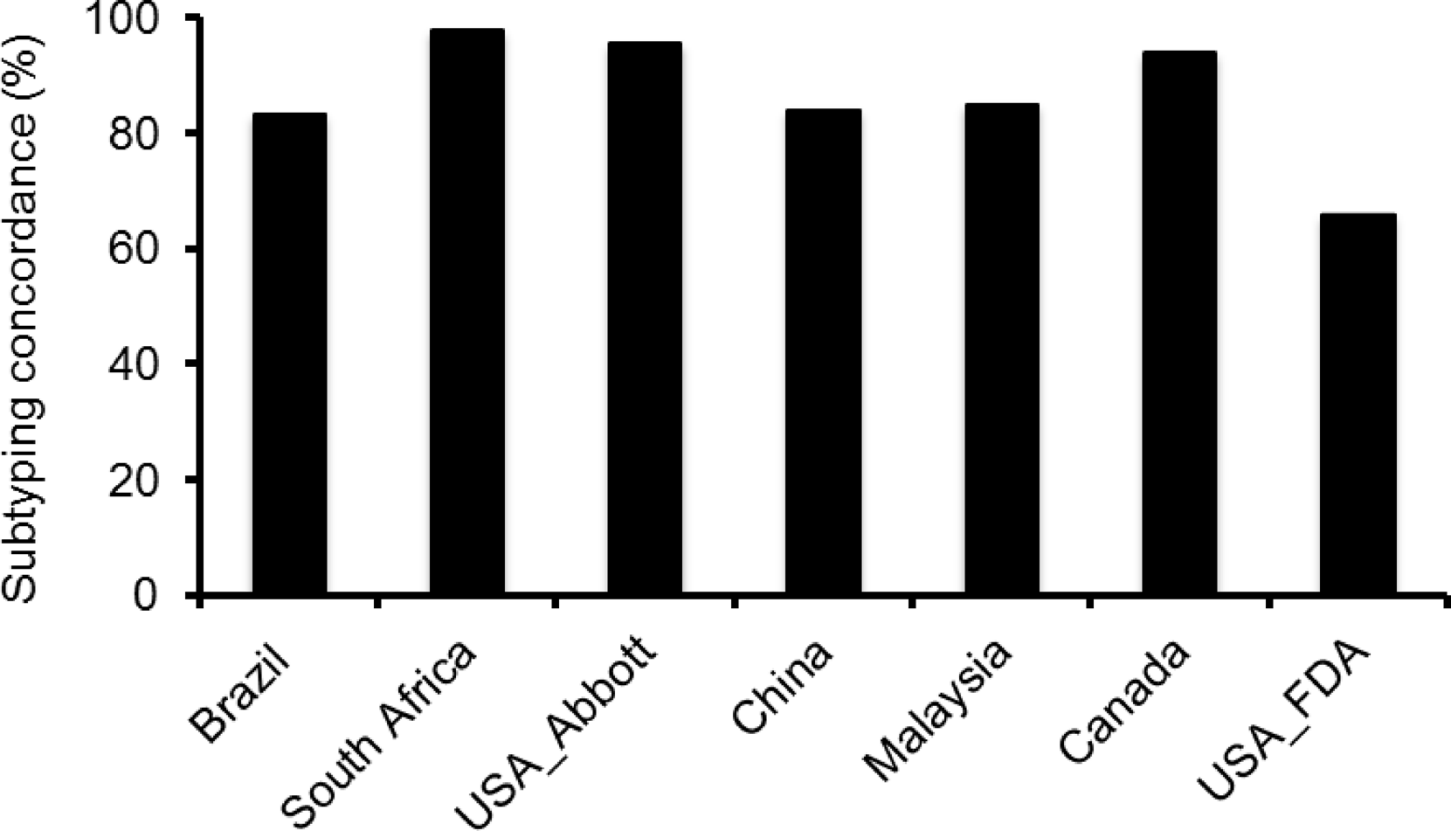
Comparison of subtype results across all sites. The subtype results from each site were compared to those determined by analyzing the 47 group M whole genome sequences at EQAPOL.

Each site used their own genetic analysis tools to determine subtypes of partial or full *pol* gene sequences. Between 1 and 7 (2.1%-14.9%) partial *pol* sequences had discordant subtype results with the EQAPOL reference sequences at South Africa, USA_Abbott, Malaysia, Canada, Brazil and China sites (Fig 2 and Table 1). All sites used one subtyping method, except the South Africa site that used three different subtyping methods (RIP, REGA and SIMPLOT) among which the results from only the REGA method (the highest concordant rate) were used for comparison. Fewer discordant subtyping results were reported for pure subtypes (6 of 168 comparisons) than recombinants (20 of 114 comparisons) in the partial *pol* gene sequences (p = 0.00032).

To investigate the causes for the discordant subtyping results, we performed additional in-depth analysis by aligning the site sequences, EQAPOL references sequences, and subtype/CRF references from the Los Alamos HIV-1 sequence database. Of the six discordant subtyping results for pure subtype viruses based on the partial *pol* sequences, two viruses had discordant results at the Brazil and Malaysia sites and one virus was discordant at the USA_Abbott and Canada sites. No discordant results were reported by the China site. Discordant result (B/D recombinant) was reported for DEMB10VE001 by the South Africa site. Analysis with two different tools (RIP and SIMPLOT) confirmed that the virus was subtype B (Table 1).

**Fig 3:**
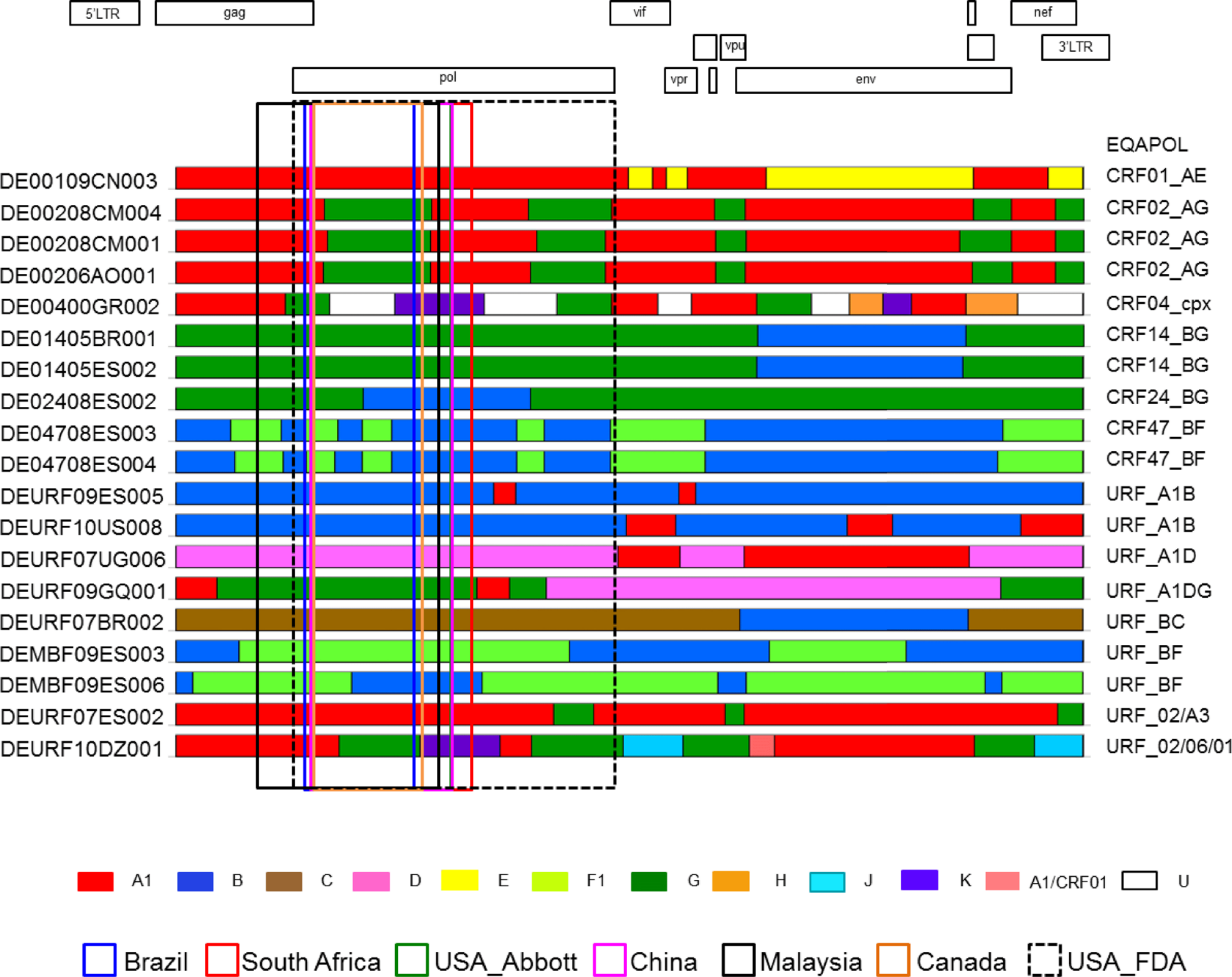
Recombination patterns in the *pol* gene and the near full length genome. The recombination patterns of near full-length sequences determined at EQAPOL are shown and indicated on the far right. The *pol* gene region amplified at each site is indicated by the colored box. Subtypes are color coded (shown at the bottom).

When the partial *pol* sequences from CRFs and URFs containing recombination breakpoints were analyzed, 20 discordant subtyping results were observed for 8 viruses (bold and underlined letters in Table 1 and Fig 3). Discordant results with 1 to 7 viruses were reported from each site. Only one discordant result was found at the South Africa and USA_Abbott sites. Four to seven discordant results were reported at the Brazil, Malaysia and China sites. Two viruses (DEURF10DZ001 and DEURF07ES002) were classified differently by five sites (Table 1), indicating that subtyping analysis can be very challenging for complex recombinants. DEURF07ES002 is an A3 sub-subtype in the sequenced region but it was classified it as A1 at the South Africa and Malaysia sites. DEURF10DZ001 is a recombinant between two CRFs, hence making the subtyping very challenging when only partial *pol* gene was sequenced (Fig 3). Five recombinants were reported as concordant with near full-length EQAPOL reference sequences by the China site, although no recombination breakpoints were present in the *pol* region sequenced for this analysis (Table 1; underlined).

**Fig 4:**
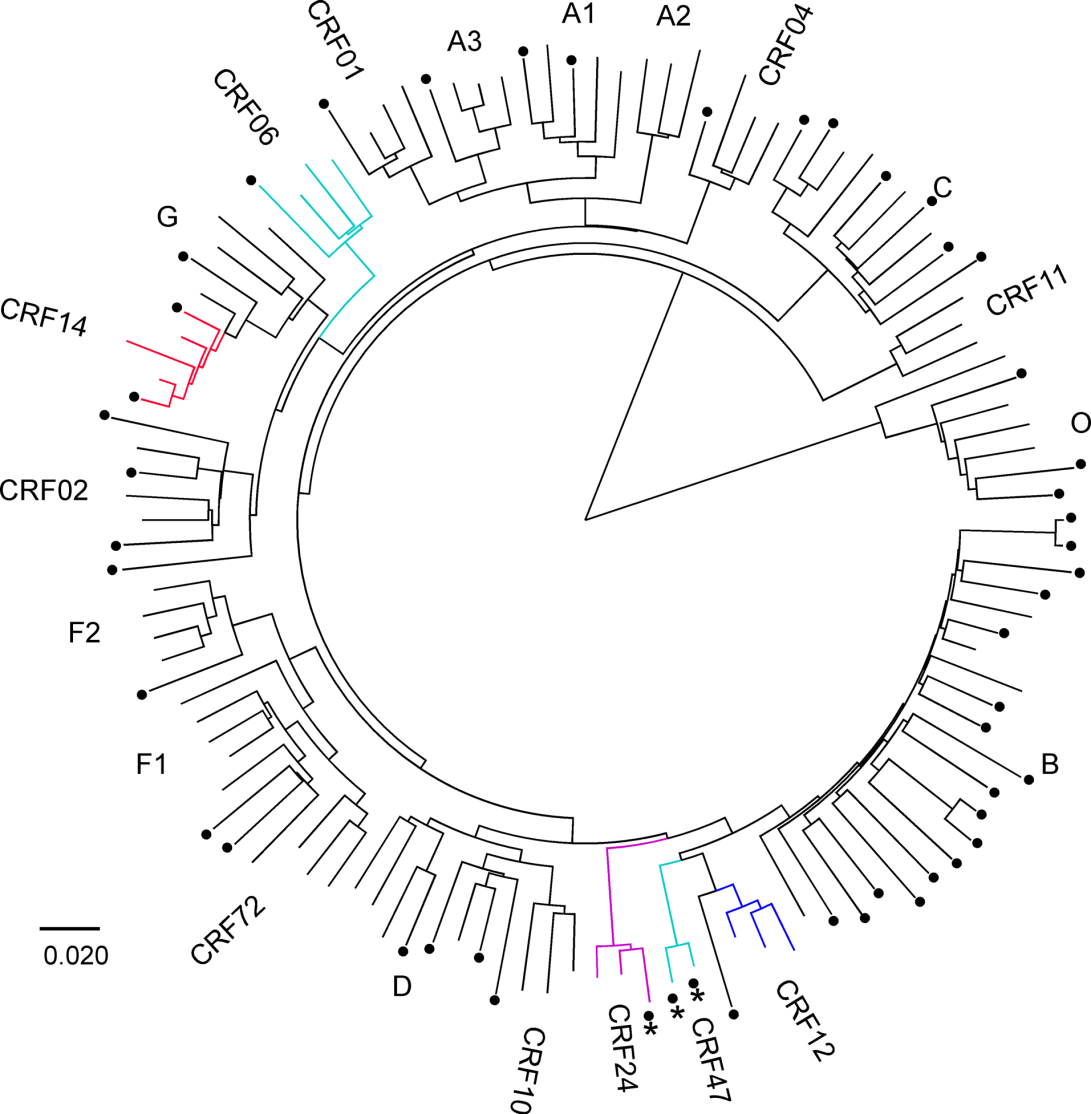
Phylogenetic clusters of CRF sequences. Partial *pol* gene sequences were aligned with all subtype references from the HIV sequence database. The phylogenetic tree was constructed using the neighbor-joining (NJ) method with Kimura two-parameter model. CRFs (06, 12, 14, 24 and 47) form distinct clusters and are depicted in different colors. The 50 sequences in the EQAPOL diversity panel are indicated by black dots. Asterisks indicate the EQAPOL sequences that are derived from the same strains used to define CRF24 and CRF47.

For CRF01 and CRF02, the unique recombination breakpoints and the pure subtype regions that have been derived from the same common ancestor can clearly distinguish them from other viruses in phylogenetic analysis. The same was observed for five sequences of CRF24_BG, CRF14_BG and CRF47_BF (Fig 4). The partial *pol* sequences from both CRF24_BG and CRF47_BF contain unique recombination breakpoints, resulting in independent clusters in the phylogenetic tree (Fig 4). One CRF24_BG and two CRF47_BF sequences in the EQAPOL diversity panel were derived from the same reference viruses used to define those two CRFs [34–36]. Consequently, they were correctly defined by the majority of the test sites (italic in Table 1) while remaining test sites correctly identified them as recombinants between the corresponding subtypes. The partial *pol* sequence of CRF14_BG viruses was pure subtype G without any recombinant breakpoints. However, phylogenetic analysis with reference sequences showed that they formed a tight cluster within the main subtype G clade (Fig 4). Thus, phylogenetic analysis with CRF14_BG reference sequences could identify this CRF in the *pol* region. Indeed, two CRF14_BG viruses were correctly identified at the USA_Abbott, China and Malaysia sites (Table 1; italic letters), while other sites correctly reported them as subtype G. These results showed that although the small partial *pol* sequences were generally reliable for subtyping pure subtypes, it was very challenging to accurately classify viruses that contain recombinant breakpoints in this region.

### Analysis of the partial *pol* gene sequences by next generation sequencing

**Fig 5:**
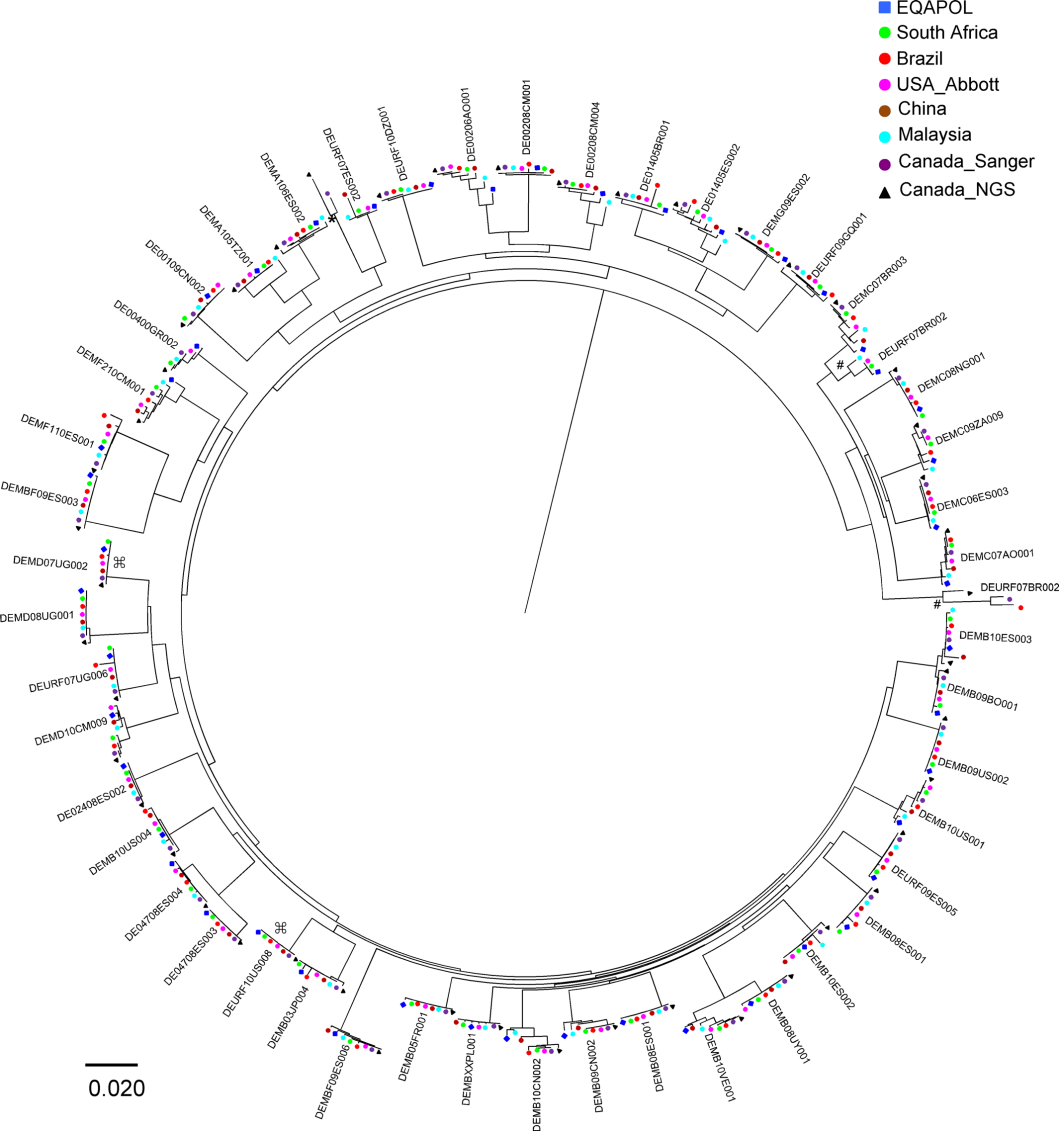
Phylogenetic analysis of partial *pol* gene sequences from all sites. All group M virus sequences (47) from six sites were analyzed together with the EQAPOL reference sequences. The phylogenetic tree was constructed using the neighbor-joining (NJ) method with Kimura two-parameter model. Sequences from the USA_FDA site were not included due to divergent sequence fragments. Ambiguous bases in DEURF07ES002 (*) and DEURF07BR002 (#) from Canada and Brazil sites resulted in independent branches. The short sequences (⌘) of DEMD07UG002 and DEURF10US008 from the Malaysia site were excluded from the analysis.

The cost and throughput of next-generation sequencing (NGS) methods have significantly improved over the last few years, and barcoded PCR amplicons derived from different samples can be batched for NGS to further reduce costs and increase the throughput [37, 38]. This makes NGS a potentially attractive alternative approach to characterize HIV-1 sequences. To investigate this possibility, the same partial *pol* PCR amplicons used for conventional Sanger sequencing were subjected to NGS analysis at the Canada site. Phylogenetic analysis of NGS consensus, generated with mixed based identification threshold at 20%, and Sanger sequences in conjunction with EQAPOL sequences from each virus showed that 37 of 47 group M viruses clustered tightly together. Since both Sanger and NGS consensus sequences reflect the dominant genotypes of HIV-1 quasispecies present in the samples, ambiguous bases were observed for both methods in nine viruses. These ambiguous bases resulted in longer branches for some viruses, or even a separate branch (DEURF07ES002) and an independent branch (DEURF07BR002) (Fig 5). A large number of ambiguous bases were also reported in DEURF07BR002 by the Brazil site and it, together with both Sanger and NGS sequences from the Canada site, formed an independent branch (Fig 5). Comparing those sequences to the EQAPOL reference sequences showed that nearly all unambiguous sites were identical to the EQAPOL references, confirming that they were indeed derived from DEURF07ES002 and DEURF07BR002, respectively.

### Analysis of the full *pol* gene sequences by next generation sequencing

The complete *pol* gene sequence was obtained by NGS at the USA_FDA site. Among all 47 group M HIV-1 isolates, 41 (87%) were successfully amplified (Fig 1 and Table 1). No PCR products were obtained for six group M viruses and all three group O viruses. Among 37 *pol* sequences that were reported to have concordant subtyping results as EQAPOL, 10 sequences from the USA_FDA site showed different subtype results (Table 1). Four other sequences showed discordant subtyping results from the EQAPOL reference sequences. Examination of *pol* sequences also showed the presence of human DNA sequences, indels, and sequence fragments that belonged to different viruses.

### All major DRMs were identified in partial *pol* sequences

Ten major DRMs were present in seven viruses (Table 2). They were all correctly identified in the partial *pol* sequences at six sites (South Africa, Brazil, China, USA_Abbott, Malaysia and Canada). Eight major DRMs were detected in 6 viruses at the USA_FDA site: Y188L in DEMB10CN002 and M46I in DEMBF09ES003 viruses were not reported. DEMB10ES003 that had major DRMs was not amplified at the USA_FDA site.

**Table 2:** Major drug resistance mutations in seven viruses.

Virus ID	PI	NRTI	NNRTI
DEMB09US002			K103N
DEMBF09ES006	M46I, I54V, L76V, V82A		K103S
DEMB10CN002	L90M	T215C	Y188L
DEMB03JP004		T215D	
DEMB10ES002	L90M		
DEMB10ES003		D67N, K219Q	
DEMB09BO001		D67N, K219Q	

In addition to the 10 major DRMs, all sites also reported some minor DRMs although they were not originally requested. While the majority of those minor DRMs were the same as identified in EQAPOL reference sequences, six (L10, K20, L33, V75, I84 and V108) were different in eight viruses (Table 3). Examination of the site sequences revealed that differences at 5 of 6 mutation sites (K20, L33, V75, I84 and V108) were due to the mixed viral population. In all five cases, mixed bases at each position were reported by the PCR product population sequencing while only single bases were reported in the SGA sequences generated at EQAPOL (Table 3). NGS methods have the potential to more accurately detect percentages of different viral species in a quasispecies population. To further validate this, we analyzed NGS sequences from the Canada site that had an average coverage of 11,987 for the polymorphism positions. A threshold of 1% was used for low abundance variant detection and reporting (Table 3). Mixtures of amino acids at V108 in DEMC07AO001 and at L33 in DEMA105TZ001 were detected and the predominant virus species were identical to the SGA sequence obtained by EQAPOL. Mixtures of amino acids at L10 in DEMBXXPL001 and also at K20 in DE0145ES002 were detected by NGS, but the EQAPOL reference sequence represented the minority species while population sequences from other test sites represented the majority virus species. No mixed populations were detected at V75 in DEMBF09ES006 and I84 in DEMF110ES001 by NGS or Sanger sequencing at any test site except Brazil, suggesting the polymorphisms at both positions may be due to *in vitro* PCR artifacts. A mixture of V and L at the L10 position in DEMBF09ES006 was detected by NGS while all other test sites only detected valine, although isoleucine was present in the EQAPOL reference sequence. Notably, the 10L phenotype in this sample was detected by NGS at frequency of 0.4%, which is considered un-reportable as 1% threshold being applied. These results confirmed that NGS analysis of amplified partial *pol* gene products could more accurately determine mixed HIV-1 populations than Sanger population sequencing, while a SGA sequence occasionally did not represent the major viral species in the HIV-1 quasispecies population of some virus stocks.

**Table 3:** Summary of discordant minor DRMs across test sites relative to the EQAPOL sequences.

Sample ID	Site	Mutations					
		PI				NRTI	
		L10I/V	K20R/I/V/L	L33I	I84I/M	V75I	V108I
DEMA105TZ001	EQAPOL	I	K	L			
	Brazil	I	K	L			
	South Africa	I	K	**I/L**^#^			
	USA_Abbott	I	K	**I**			
	China	I	K	L			
	Malaysia	**V**	**R**	L			
	Canada (Sanger)	I	K	**I**			
	Canada (NGS)	I (97.2%), L (2.8%)	K (100%)	**I** (19.7%), L (80.3%)			
DEMB10CN002	EQAPOL		V				
	Brazil		**V/L**^$^				
	South Africa		**V/L**^$^				
	USA_Abbott		**V/L**^$^				
	China		L				
	Malaysia		L				
	Canada (Sanger)		**V/L**^$^				
	Canada (NGS)		**V** (35.8%) **L** (64.2%)				
DEMBXXPL001	EQAPOL	L					
	Brazil	Neg PCR					
	South Africa	**V**					
	USA_Abbott	**V**					
	China	L					
	Malaysia	L					
	Canada (Sanger)	**V**					
	Canada (NGS)	**V** (65.5%), L (33.5%)					
DEMC07AO001	EQAPOL						V
	Brazil						I/V*
	South Africa						I
	USA_Abbott						V
	China						V
	Malaysia						V
	Canada (Sanger)						I/V*
	Canada (NGS)						**I** (23.0%), V (77.0%)
DEMF110ES001	EQAPOL				I		
	Brazil				I/M*		
	South Africa				I		
	USA_Abbott				I		
	China				I		
	Malaysia				I		
	Canada (Sanger)				I		
	Canada (NGS)				I (100%)		
DE01405ES002	EQAPOL		V				
	Brazil		**I**				
	South Africa		**I**				
	USA_Abbott		**I**				
	China		**I**				
	Malaysia		**I**				
	Canada (Sanger)		**I**				
	Canada (NGS)		**I** (96.3%)**, V** (3.7%)				
DEMBF09ES006	EQAPOL	I				V	
	Brazil	**V**				I/V*	
	South Africa	**V**				V	
	USA_Abbott	**V**				V	
	China	**V**				V	
	Malaysia	**V**				V	
	Canada (Sanger)	**V**				V	
	Canada (NGS)	**V** (99.6%), L(0.4%)				V (100%)	
DEURF07BR002	EQAPOL	L					
	Brazil	L					
	South Africa	L					
	USA_Abbott	L					
	China	L					
	Malaysia	**F**					
	Canada (Sanger)	L					
	Canada (NGS)	L (100%)					

Discordant DRMs between each site and EQAPOL sequences are indicated in bold. Percentages of mutations determined by NGS at the Canada site are indicated in parenthesis. The positions are shown relative to those in the HXB2 genome.

*: a mixture of G and A

#: a mixture of T and A

$: a mixture of G and C

Analysis of complete *pol* sequences generated by NGS at the FDA site showed different patterns of DRMs. In addition to D67N and K219Q in DEMB09BO001 and K103N in DEMB09US002 that were detected by all sites, a minor A71I mutation was also reported for both viruses, although examination of the sequences did not show the A71I mutation in either sequence. Among five major DRMs in DEMBF09ES006 (Table 2), four were reported while the M46I mutation was not detected in the sequence due to A to G substitution in the sequence from the site.

## Discussion

Determination of subtypes and recombinants of HIV-1 and monitoring of DRMs are routinely performed in many research and national reference labs globally [14, 39, 40]. The partial *pol* gene is an advantageous target region for molecular surveillance since the sequences can be used for both subtyping and drug resistance analysis [41]. However, the target region, RT-PCR conditions utilized, genetic analysis tools and choice of reference sequences vary from site to site. Our analysis of results from seven international sites showed that the majority of 50 genetically diverse HIV-1 isolates were successfully amplified and correctly subtyped. However, optimization and standardization of HIV-1 genotyping methods would improve success rates of RT-PCR amplification and achieve more accurate subtype assignment.

All 47 group M viruses were amplified by RT-PCR at the South Africa, USA_Abbott, Malaysia and Canada sites, while 89.4%-94.0% of these viruses were amplified at the Brazil and China sites. Since all group M viruses were successfully amplified in a single amplicon at South Africa, USA_Abbott and Canada sites, the primers and conditions used at these sites with a 100% PCR amplification rate are recommended for global subtype and drug resistance surveillance. The same commercial kit was used at both the USA_Abbott and China sites (S1 Table). However, 100% PCR success rate was reported at the USA_Abbott site but only 84.1% at the China site. This suggests the need for optimization and standardization of operation procedures as well as standard personnel training among different sites. In this study only high viral genome copy samples (10^7^/ml) were used to ensure that the failure of amplification of HIV-1 *pol* gene or whole genome was not due to the low copy numbers in the samples. Thus, this study did not address the issues that might occur with samples that had low copy viral genomes during screening, diagnosis and follow-up. The PCR sensitivity for samples with low viral genome numbers among all test sites will be addressed in a separated study. When the complete *pol* gene sequence was analyzed by NGS, a lower success rate (87%) was observed, indicating that a larger genome amplification (~3000bp) and subsequent NGS analysis by this method may not be practical. However, adoption of newer methods for library preparation such as HIV-SMART and metagenomics NGS sequencing will advance the field and likely supplant traditional sub-genomic PCR and standard sequencing in the future [42, 43].

While the majority of the subtype classifications based on partial *pol* gene sequences were accurate for pure subtypes, determination of recombination patterns and hence classification of CRFs and URFs were considerably more challenging. Among 168 comparisons for the pure subtype sequences in the partial *pol* gene, only 6 had discordant results compared to the EQAPOL references. When the same partial *pol* gene sequences with recombination breakpoints were analyzed, the recombinant sequence regions for each subtype were smaller, making subtype assignment more difficult. However, pure subtype sequences represented in CRFs are generally derived from one common ancestor or have evolved into closely related sequences to form tight clusters in phylogenetic analysis. In such cases, they could be accurately classified even with relatively small partial *pol* gene sequences (for example, CRF12, CRF14, CRF24 and CRF47 in Fig 4). Since the subtyping results based on partial *pol* gene sequences could not predict the complex recombinant nature of viruses in the non-sequenced regions, the recombination patterns of such recombinants could only be fully resolved by analyzing whole genome sequences.

Group O viruses, as expected, were not amplified with group M virus-specific primers. Only the Malaysia site analyzed the panel viruses with group O specific primers and successfully amplified all three group O viruses, according to their standard protocol for samples that fail to amplify with group M primers. The conventional global subtyping and drug resistance survey with group M specific primers will therefore miss viruses of other groups. While this might not significantly affect the survey results in areas where group M viruses are predominant, non-group M primers should be included in areas where epidemics may include other group viruses, especially when PCR targeting group M viruses is unsuccessful.

All 10 DRMs in seven isolates were detected by all six sites where the partial *pol* sequences were analyzed. This is most likely because these mutations were present in the predominant virus populations (>97% of NGS reads as reported by the Canada site). However, the ability to detect mixed bases at the minority DRM sites varied among samples. This indicates that if the major DRMs are present as minority populations in samples, their presence might not be reliably detected by SGA or by conventional Sanger sequencing of PCR products. However, the percentages of variants at those positions could be more accurately determined by NGS analysis of the same partial *pol* PCR amplicons. With significant reduction of cost using the primer ID technology, NGS methods could be a good alternative tool for global surveillance when the resources are available. Importantly, the higher sensitivity of NGS for detection of minor DRMs relative to Sanger population sequencing will allow surveillance of minority DRMs in the population.

More diverse HIV-1 isolates continue to be characterized by EQAPOL, including other HIV-1 group viruses and HIV-2 isolates. A more comprehensive panel that fully represents HIV-1 groups, subtypes, major CRFs as well as HIV-2 can be assembled in the future to more rigorously evaluate accuracy and efficiency of global surveillance tools. NGS analysis of partial *pol* sequences showed promise in subtyping and detection of DRMs, whereas analysis of larger sequences by NGS demonstrated poorer performance. With continuous advances and optimization, it is likely that NGS analysis of whole HIV-1 genome from clinical samples will be refined to more accurately assign subtypes and detect DRMs for all classes of antiretroviral drugs including those that do not target the protease and reverse transcriptase genes.

## Supporting Information

S1 Table Summary of the PCR conditions and sequence analysis methods in all seven international sites. (PDF)
