# Supplementary material for: Genetic Characterization of a Panel of Diverse HIV-1 Isolates at Seven International Sites
Source: PLoS One. 2016 Jun 17;11(6):e0157340. doi: 10.1371/journal.pone.0157340 (PMC4912073; doi:10.1371/journal.pone.0157340)
Supplement: S1 Table — (PDF) [file pone.0157340.s001.pdf]

S1 Table. Summary of the PCR conditions and sequence analysis methods in all seven international sites.

| Methods | EQAPOL | Brazil | South Africa | USA_Abbott | China | Malaysia | Canada | USA_FDA |
|---|---|---|---|---|---|---|---|---|
| Amplified region | Near full-length genome | Partial *pol* gene | Partial *pol* gene | Partial *pol* gene | Partial *pol* gene | Partial *pol* gene | Partial *pol* gene | Complete *pol* gene |
| Amplification method | Nested PCR | Nested PCR | In house method using Expand High Fidelity PLUS PCR System (Roche) | ViroSeq™ HIV-1 RT-PCR Module, v2 | ViroSeq™ HIV-1 RT-PCR Module, v2 | Two independent nested PCR (PR and RT) | One-step RT-PCR plus nested PCR using in-house protocol | One-step RT-PCR |
| Subtyping method | REGA, jPHHM | Stanford database | REGA, RIP, SIMPLOT | BioEdit, v7.0.4.1<br>Phylip, v3.573<br>SimPlot, v3.5.1 | BioEdit, v7.0.4.1<br>HIV database | HIV database | REGA | HIVE |
| Phylogenetic tree method | Neighbor-joining | Not Done | Neighbor-joining | Neighbor-joining | Neighbor-joining | Neighbor-joining | Neighbor-joining | Neighbor-joining |
| Length of amplicon | Two overlapping single genome amplicons (~4400bp and 4700bp) | ~1700bp | ~1770bp | ~1302bp | ~1302bp | ~1600bp (1200bp if optional primer used) | ~1084 bp | ~3000bp |
| Software package for consensus generation | Sequencher v5.1 | | Sequencher v5.0 | ViroSeq HIV-1 Genotyping System Software | ViroSeq HIV-1 Genotyping System Software | DNASIS | ReCALL for Sanger Sequencing and HyDRA for NGS | HIVE |
| Detection of DRMs | Stanford database | Stanford database | Stanford database | ViroSeq HIV-1 Genotyping System Software, v2.8 | ViroSeq HIV-1 Genotyping System Software, v2.8 | Stanford database | Stanford database | Stanford database |
| Reverse transcription | 65°C for 5 min<br>4°C for 1 min<br>Add SuperScript III<br>55°C for 1 hr<br>70°C for 15 min | 70°C for 10 min<br>42°C for 5 min<br>Add RT mix<br>42°C for 1 hr 30 min<br>70°C for 10 min | In-house method using Thermoscript TM RT-PCR System<br>65°C for 5 mins<br>85°C for 1 min<br>Add RT mix<br>55°C for I hr<br>85°C for 5 mins<br>Hold 4°C | 42°C for 60min<br>99°C for 5min<br>Hold; 4°C at least 10min | 65°C for 30sec<br>42°C for 5min<br>Add RT mix<br>42°C for 60min<br>99°C for 5min<br>Hold at 4°C at least 10min | 65°C for 5min<br>Snap chill on ice for 1min<br>Add RT mix<br>25°C for 5min<br>50°C for 60min<br>70°C for 15min | see to "1st round PCR" below | NA |
| One step RT-PCR | NA | NA | NA | NA | NA | NA | NA | 1 cycle; 50°C for 30min<br>1 cycle; 94°C for 2 min<br>35 cycles;<br>94°C for 20 sec<br>50°C for 30sec<br>68°C for 1min 30 sec<br>1 cycle; 68°C for 5min<br>Hold at 4°C to end |
| 1st round PCR | 1 cycle; 94°C for 2 min<br>35 cycles;<br>94°C for 15 sec,<br>60°C for 30 sec<br>68°C for 4 min and 30 sec<br>1 cycle; 68° C for 10 min | 1 cycle; 94°C for 1 min<br>35 cycles;<br>94°C for 45 sec,<br>55°C for 45 sec<br>72°C for 2 min<br>1 cycle; 72° C for 10 min | 1 cycle; 94°C for 5 min<br>35 cycles;<br>94°C for 20 sec,<br>50°C for 30 sec<br>72°C for 2 min<br>1 cycle; 72° C for 2 min | 1 cycle; 50°C for 10min<br>1 cycle; 93°C for 12min<br>40 cycles;<br>93°C for 20sec,<br>64°C for 45sec,<br>66°C for 3min<br>1 Cycle; 72°C for 10min<br>Hold; 4°C | 1 cycle; 50°C for 10min<br>1 cycle; 93°C for 12min<br>40 cycles;<br>93°C for 20sec,<br>64°C for 45sec,<br>66°C for 3min<br>1 Cycle; 72°C for 10min<br>Hold; 4°C | 1 cycle; 95°C for 5 minutes<br>35 cycles;<br>94°C for 30 sec<br>55°C for 30 sec<br>72°C for 1 min and 15 sec<br>1 cycle; 72°C for 10 minutes | 1 cycle; 50°C for 30min<br>1 cycle; 94°C for 2 min<br>40 cycles;<br>94°C for 20 sec<br>50°C for 30sec<br>68°C for 1min 30 sec<br>1 cycle; 68°C for 5min<br>Hold at 4°C to end | NA |
| 2nd round PCR | 1 cycle; 94°C for 2 min<br>35 cycles;<br>94°C for 15 sec,<br>60°C for 30 sec<br>68°C for 4 min and 30 sec<br>1 cycle; 68° C for 10 min | 1 cycle; 94°C for 1 min<br>35 cycles;<br>94°C for 1 min<br>55°C for 1 min<br>72°C for 1 min and 30 sec<br>1 cycle; 72º C for 10 min | 1 cycle; 94°C for 5 min<br>35 cycles;<br>94°C for 20 sec,<br>50°C for 30 sec<br>72°C for 2 min<br>1 cycle; 72° C for 2 min | NA | NA | 1 cycle; 95°C for 5 minutes<br>35 cycles;<br>94°C for 30 sec<br>55°C for 30 sec<br>72°C for 1 min and 15 sec<br>1 cycle; 72°C for 10 minutes | 1 cycle; 98°C for 30sec;<br>35 cycles;<br>98°C for 10 sec,<br>62°C for 20sec,<br>72°C for 40 sec<br>1 cycle; 72°C for 10min<br>Hold at 4°C to end | NA |

**Primers used at EQAPOL**
Reverse transcription 5' half: 07Rev9 5'-CTTCCTGCCATAGGAGATGCCTAA-3'
Reverse transcription 3' half: 1.R3.B3R 5'-ACTACTTGAAGCACTCAAGGCAAGCTTTATTG-3'
1st Round 5' half: 1.U5.B1F 5'-CCTTGAGTGCTTCAAGTAGTGTGTGCCCGTCTGT - 3' and 07Rev8 5 '-CCTARTGGGATGTGTACTTCTGAACTT -3'
1st Round 3' half: 07For7 5'-CAAATTAYAAAAATTCAAAATTTTCGGGTTTATTACAG-3' and 2.R3.B6R 5'-TGAAGCACTCAAGGCAAGCTTTATTGAGGC-3'
2nd Round 5' half: Upper1A 5'- AGTGGCGCCCGAACAGG-3' and Rev11 5'-ATCATCACCTGCCATCTGTTTTCCAT-3'
2nd Round 3' half: VIF1 5'-GGGTTTATTACAGGGACAGCAGAG-3' and Low2c 5'-TGAGGCTTAAGCAGTGGGTTCC-3'

**Primers used at the Brazil site**
1st Round: Kozal 1 5'- CAGAGCCAACAGCCCCACCA - 3' and Kozal 2 5 '- TTTCCCCACTAA CTTCTGTATGTCATTGA-3'
2nd Round: DP10 5'-TAACTCCCTCTCAGCAGAAGGAGCCG - 3' and FRENKEL 2 IV (inner) 5 '- GTATGTCATTGACAGTCCAGC -3'
Optional primer: DP1 5 '-CCTCAAATCACTCTTTGGCAAC - 3' and RT4 5 '- AGTACTTAACCCATCCAAAG – 3'

**Primers used at the South Africa site**
1st Round: G25REV 5'-GCAAGAGTTTTGGCTGAAGCAATGAG- 3' and IN3 5'-TCTATVCCATCTAAAAATAGTACTTTCCTGATTCC- 3'
2nd Round: AV150 5'-GTGGAAAGGAAGGACACCAAATGAAAG-3' and PolM4 5'-CTATTAGCTGCCCCATCTACATA-3'

**Primers used at the USA_Abbott site**
List 4J94-91: Forward & Reverse Primers

**Primers used at the China site**
List 4J94-91: Forward & Reverse Primers

**Primers used at the Malaysia site**
1st Round PR gene: 507A 5'-AAGGAACCCTTTAGAGACTATGTAGA-3' and 503B 5'-TATGGATTTTCAGGCCCAATTTTTG-3'
1st Round RT gene: K1 5'-GGAAACCAAAAATGATAGGGGGAATTGGAGG-3' and 326B 5'-CTGTACTTCTGCTACTAAGTCTTTTGATGGG-3'
2nd Round PR gene: 508A 5'-GTAAAAAATTGGATGACAGAAACCTTG-3' and 504B 5'-ACTTTTGGGCCATCCATTCC-3'
2nd Round RT gene: K3 5'-GTGGAAAAAAGGCTATAGGTACAG-3' and 328B 5'-CTGCCAACTCTAATTCTGCTTC-3'
1st Round Group O: DRPR05F 5'-AGACAGGYTAATTTTTTAGGGA-3' and 503B 5'-TATGGATTTTCAGGCCCAATTTTTG-3'
2nd Round Group O: DRPR01M 5'-AGAGCCAACAGCCCCACCAG-3' and 504B 5'-ACTTTTGGGCCATCCATTCC-3'

**Primers used at the Canada site**
1st Round: F 5'-GARAGACAGGCTAATTTTTTAGGGA-3' and R 5'-ATCCCTGCATAAATCTGACTTGC-3''
2nd Round: F 5'-CTTTARCTTCCCTCARATCACTCT-3' and R 5'-CTTCTGTATGTCATTGACAGTCC-3

**Primers used at the USA_FDA site**
Q: 5'-GTTTCAATTGTGGCAAAGAAGGGC-3' and vifcr: 5'-TTGGTCTTCTGGGGCTTGTTC-3'
